# Catastrophe risk can accelerate unlikely evolutionary transitions

**DOI:** 10.1098/rspb.2021.2711

**Published:** 2022-03-30

**Authors:** Andrew E. Snyder-Beattie, Michael B. Bonsall

**Affiliations:** Mathematical Ecology Research Group, Department of Zoology, University of Oxford, Oxford OX1 3PS, UK

**Keywords:** astrobiology, anthropic principle, evolution, major transitions, punctuated equilibrium

## Abstract

Intelligent life has emerged late in Earth’s habitable lifetime, and required a preceding series of key evolutionary transitions. A simple model (the Carter model) explains the late arrival of intelligent life by positing these evolutionary transitions were exceptionally unlikely ‘critical steps’. An alternative model (the neocatastrophism hypothesis) proposes that intelligent life was delayed by frequent catastrophes that served to set back evolutionary innovation. Here, we generalize the Carter model and explore this hypothesis by including catastrophes that can ‘undo’ an evolutionary transition. Introducing catastrophes or evolutionary dead ends can create situations in which critical steps occur rapidly or in clusters, suggesting that past estimates of the number of critical steps could be underestimated. If catastrophes affect complex life more than simple life, the critical steps will also exhibit a pattern of acceleration towards the present, suggesting that the increase in biological complexity over the past 500 Myr could reflect previously overlooked evolutionary transitions. Furthermore, our results have implications for understanding the different explanations (critical steps versus neo-catastrophes) for the evolution of intelligent life and the so-called Fermi paradox—the observation that intelligent life appears rare in the observable Universe.

## 1. Introduction

Intelligent life has emerged late in Earth’s lifetime. In about 1 billion years (Gyr), the increasing luminosity of the Sun in its later stages of life will destroy the Earth’s ability to support complex life [1,2], a comparatively short amount of time compared to the 4 Gyr that it has taken for intelligence to emerge. The fact that intelligent life emerged on a time scale within an order of magnitude of our star’s lifetime is puzzling, as the time scales associated with biological and stellar evolution are driven by different processes and thus ought to be uncorrelated.

This coincidence was first noticed by Carter [3], who proposed a resolution to the puzzle based on observation selection effects. Anthropic principles (such as observation selection effects) occur when some property (such as the evolution of intelligence) is correlated with the observer existing in the first place [3,4]. Carter argued that, if *L* denotes the habitable lifetime of Earth, and *τ* is the time scale it takes for evolution to produce intelligent life, and these time scales differ by many orders of magnitude, then we rule out the possibility that *τ* ≪ *L*; intelligent life did not emerge in the early stages of Earth’s lifetime. However, due to observation selection effects, we can not rule out that *τ* ≫ *L*; the possibility that intelligent life typically takes much longer than Earth’s lifetime. Although most biospheres will expire long before intelligent life emerges, for the rare instances in which it does emerge, such life will inevitably still find itself emerging within the habitable lifetime of its environment. Moreover, this emergence will occur on roughly the same time scale as the environment’s lifetime, consistent with our observation of *τ* ≈ *L*. This reasoning has become known as the Carter argument.

There could be a number of reasons for why intelligence in general takes a long time to emerge or is a rare event. On Earth, intelligent life required a preceding series of evolutionary transitions [5], such as abiogenesis, eukaryogenesis, and the emergence of sexual reproduction, multicellular life and intelligence itself. Although some of these transitions such as multicellularity have occurred multiple times throughout Earth’s history [6], other transitions have only occurred once and may have been exceptionally unlikely, even in conducive environments. Built around Carter’s original argument, studies have examined models where a small number of rare ‘critical steps’ are required for intelligent life [7–12]; where a *critical step* is defined as one that has an exceptionally low probability per unit time, so that its expected time greatly exceeds the lifetime of Earth.

As Carter’s model predicts, on average, evenly spaced critical steps, this can be used to evaluate whether a particular evolutionary transition is likely to be a critical step. If a transition happens quickly or a cluster of transitions occurs in a short period of time, this model would predict that the rapid transitions would not be a critical step. The rapid origin of life has been cited as possible evidence that it is not a critical step [13], and subsequent analysis using the critical step model predicted that some of the key evolutionary transitions related to the origin of life, as defined [5] (emergence of replicating molecules, chromosomes and the central dogma of molecular biology), could not have been critical steps as all three occurred within the first few hundred million years of Earth’s history [9]. Similarly, despite the increase in biological complexity over the past 500 Myr, the even spacing property of the Carter model has led to the conclusion that at most one or two critical steps could have occurred in this time period [7].

Similar to the Carter argument, the neocatastrophism hypothesis [14] seeks to explain why we do not see any signs of other intelligent civilizations in our galaxy, despite the hundreds of billions of stars that could potentially be hosts to habitable planets [15]. Many of the conditions for habitable planets in our galaxy seem to have been in place many billions of years ago [16], suggesting that if intelligent life is common, we should expect to find evidence of many civilizations in our galaxy far older than our own. The neocatastrophism hypothesis argues that our failure to observe ancient intelligent civilizations could still be reconciled with intelligent life being common, if intelligent life were just now emerging simultaneously across our galaxy. This correlation in emergence times for intelligent life could arise if intelligent life was suppressed beforehand by galaxy-wide catastrophes (such as gamma-ray bursts) [14,17]. If intelligent life requires a period of catastrophe-free time to emerge successfully, then we may expect intelligent life to emerge in a correlated fashion across the galaxy in the first sufficiently long window of catastrophe-free time. The neocatastrophism hypothesis then predicts that intelligent life will take a long period of time to emerge successfully on Earth, in a similar way as the Carter argument. However, the hypotheses differ greatly in their proposed resolutions to the apparently lifeless galaxy. The neocatastrophism hypothesis resolves the question by suggesting that intelligent life is just getting started across the galaxy and is not yet detectable, while the Carter argument predicts that intelligent life required a small set of exceptionally rare critical steps, and thus is itself exceptionally rare.

Here, we generalize the critical steps model by allowing for catastrophes that ‘undo’ evolutionary transitions, as well as allowing for niche incumbency effects (local temporal states that limit evolutionary and/or ecological expansion e.g. [18]) through evolutionary ‘dead ends’, that could preclude a critical step. We show that these conditions can result in unevenly spaced critical steps, including critical steps that occur rapidly, in tight clusters, or that accelerate over time. In addition to opening up new hypotheses about whether the number of critical steps has been underestimated, this work also enables us to test the Carter argument and the neocatastrophism hypothesis.

## 2. Methods

Here, we assume that intelligent life requires *n* evolutionary transitions, each of which is exponentially distributed with a constant rate *λ*_*i*_. We assume that the transitions must occur in sequence, so that the second transition can only occur after the first, the third after the second and so on. This can be modelled as a continuous-time Markov chain, for which a substantial amount of theory already exists [19]. Let the Markov chain be denoted *X*(*t*) with *n* + 1 states *i* = 0, 1, …, *n*, where the state *i* = 0 is the state before the first evolutionary transition and the *i*th state represents the time after the *i*th transition but before the next one. We assume *X*(0) = 0, so that Earth starts with no evolutionary transitions having occurred. Let *q*_*ij*_ indicate the rate at which the chain moves from state *i* to state *j*, producing a generator matrix **Q**:

2.1 $$Q=\left[\begin{array}{cccc} -q_{0} & q_{01} & \ldots & q_{0n}\\ q_{10} & -q_{1} & \ldots & q_{1n}\\ \vdots & \vdots & \ddots & \vdots \\ q_{n0} & q_{n1} & \ldots & -q_{n} \end{array}\right]$$

where $q_{i}=\sum_{i\neq j}q_{ij}$, so that all rows sum to 0. In the case of *n* evolutionary transitions occurring at rate *λ*_*i*_, our generator matrix is:

2.2 $$Q=\left[\begin{array}{ccccc} -\lambda_{1} & \lambda_{1} & 0 & \ldots & 0\\ 0 & -\lambda_{2} & \lambda_{2} & & \vdots \\ \vdots & \ddots & \ddots & \ddots & 0\\ 0 & \ldots & 0 & -\lambda_{n} & \lambda_{n}\\ 0 & \ldots & 0 & 0 & 0 \end{array}\right],$$

where the final row of zeros indicate that the *n*th state is an absorbing state, representing the emergence of intelligent life. This reflects our interest in studying the transient behaviour of *X*(*t*) before intelligence emerges, rather than an assumption that no further evolutionary transitions are possible once intelligent life emerges.

We provide two modifications to this basic model. The first variation is to allow some states in the chain to transition to earlier states, representing catastrophes or other setbacks that could ‘undo’ evolutionary transitions. The second variation is to create additional absorbing states that represent evolutionary ‘dead ends'. We define *μ* to be the catastrophe rate, and *θ* to be the evolutionary transition rate to an evolutionary dead end. Illustrations of Markov chains with these characteristics are given in table 1.

**Table 1.** A diagram of the Markov chain models for a catastrophe model and an evolutionary dead end model, along with their transition matricies.

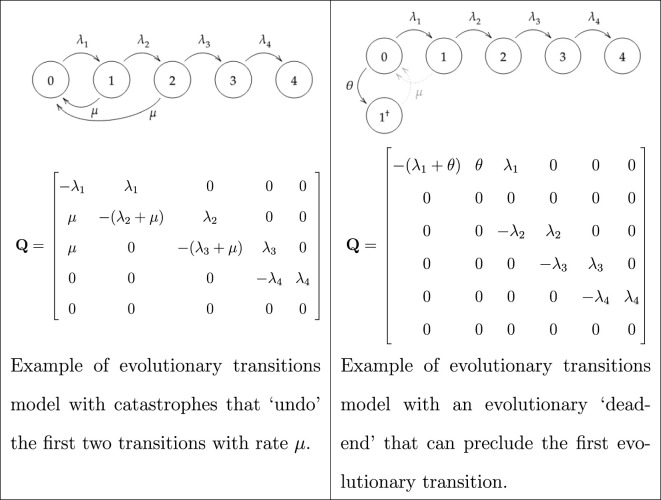

By varying the structure and rates of *X*(*t*), we can investigate different hypotheses about evolutionary transitions. To incorporate observation selection effects, we condition all probabilities on the eventual emergence of intelligent life before the end of Earth’s lifetime. Let *L* denote the habitable lifetime of Earth. Our goal is to calculate *P*(*X*(*t*) = *i*|*X*(*L*) = *n*) for all *i*, the state probabilities throughout Earth’s history conditional on intelligence (the final transition) emerging before the end of Earth’s lifetime. Using Bayes's rule, we obtain

2.3 $$P(X(t)=i|X(L)=n)=\frac{P(X(L)=n|X(t)=i)\;P(X(t)=i)}{P(X(L)=n)}.$$

Let *π*_*i*_(*t*) be defined as *P*(*X*(*t*) = *i*), the probability of being in the *i*th state at time *t* and ***π***(*t*) be the row vector of these state probabilities $\pi (t)=[\pi_{0}(t)\;\pi_{1}(t)\;\ldots \;\pi_{n}(t)]$. Following standard Markov chain theory (e.g. [19]), the state probabilities can be calculated using the equation

2.4 π(t)=π(0)P(t),

where the matrix **P**(*t*) is found from the Kolmogorov backward equation

2.5 $$P^{\prime }(t)=QP(t),$$

where the entries of **P**(*t*) are *p*_*ij*_(*t*), denoting the probability of moving from state *i* to state *j* within time *t*, and the entries of **P**′(*t*) are the derivative of each *p*_*ij*_(*t*) with respect to time. Let $\pi_{i}^{⋆}(t)$ denote *P*(*X*(*t*) = *i*|*X*(*L*) = *n*), the probability of being in state *i* at time *t* conditional on the final transition occurring before the end of Earth’s lifetime. The calculation for each $\pi_{i}^{⋆}(t)$ can be written using Bayes's rule,

2.6 $$\pi_{i}^{⋆}(t)=p_{in}(L-t)p_{0i}(t)p_{0n}^{-1}(L),$$

where the first term is the probability that the process moves from state *i* to state *n* in the remaining time left on Earth, *L* − *t*, the second term denotes the probability of having moved from the initial condition of state 0 to state *i* within time *t*, and the final term is a normalizing constant denoting the probability that the final transition is reached by the end of Earth’s lifetime.

When the generator matrix **Q** is constant in time, the solution of the Kolmogorov backward equations takes the form of a matrix exponential,

2.7 $$P(t)=e^{Qt}=\sum_{k=0}^{\infty }\frac{t^{n}}{n!}Q^{n}.$$

However, in order to test the neocatastrophism hypothesis, we also want to investigate what happens if the catastrophe rate declines over time, producing a system of differential equations of the form **P**′(*t*) = **Q**(*t*)**P**(*t*) that lacks a closed form solution. We instead adopt a numerical integration scheme from [20] based on an explicit Runge–Kutta method with adaptive step size control.

Catastrophes are modelled by allowing the Markov chain to revert to an earlier state. This undoing of preceding evolutionary transitions allows different hypotheses (i.e. critical step with catastrophes, niche incumbency, neocatastrophism) to be investigated depending which transitions are vulnerable and how many transitions are undone (see table 1; electronic supplementary material for further details). The code for our models is available at https://osf.io/4xrb9/.

## 3. Results

**Figure 1.**
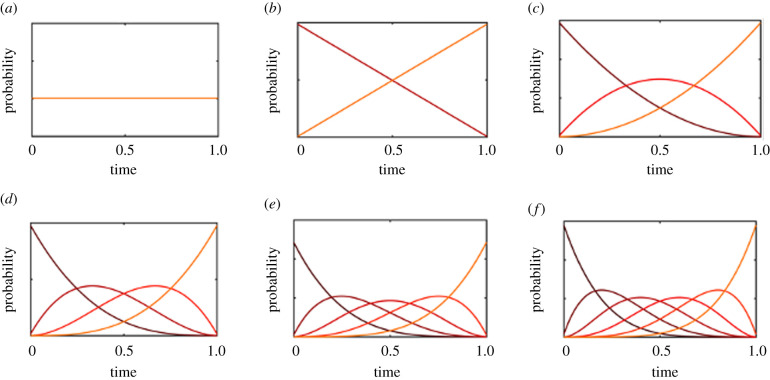
Probability distributions for the timing of each critical step, in a model with between (*a*) one and (*f*) six critical steps, with each expected transition time greatly exceeding the lifetime of Earth. The steps are evenly spaced out across Earth’s lifetime, identical to the results by [9,10]. (Online version in colour.)

We begin by evaluating the simplest model with *n* evolutionary transitions, no catastrophes, and no evolutionary dead ends. We normalize the lifetime of Earth to *L* = 1, and set all the transition rates to *λ*_*i*_ = 10^−3^, so that the expected time for each transition greatly exceed the habitable lifetime of Earth by three orders of magnitude. In doing so, this model corroborates existing results, in producing probability distribution functions for each critical step that are evenly spaced throughout the habitable lifetime of the planet (figure 1). For a model with one critical step, the probability of the transition occurring at a particular time becomes uniform over the habitable interval. As the number of critical steps increases, the probability distributions for each transition (with associated means and higher order moments) become compressed and evenly spaced, so that the *k*th critical step should occur approximately at the point *k*/(*n* + 1) in the planet’s lifetime.

**Figure 2.**
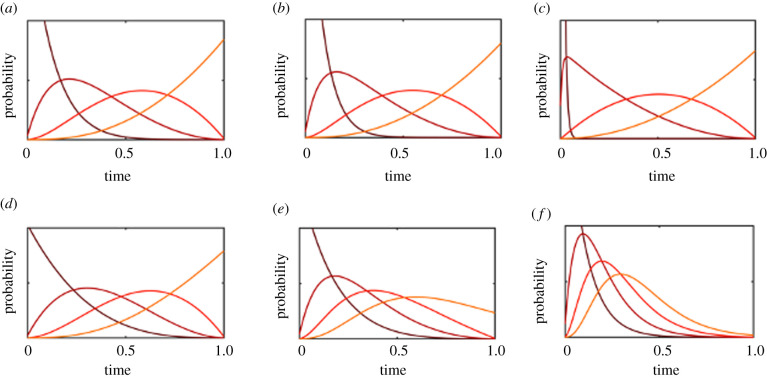
Probability density functions for transition times with faster rates in a four step model. In the first row, only the first transition becomes faster, starting with *λ*_1_ = 5 (*a*), then moving to *λ*_1_ = 10 (*b*) and *λ*_1_ = 100 (*c*). In the second row, all transitions become faster, starting with *λ* = 1 (*d*), then moving to *λ* = 5 (*e*) and *λ* = 10 (*f*). (Online version in colour.)

We can also examine what happens when a transition rate becomes more rapid, so that the expected transition time occurs within Earth’s lifetime (figure 2*a*–*c*). Unsurprisingly, faster transitions produce probability density functions that are skewed towards earlier transitions. When the rate is fast enough, a transition will occur almost immediately after the preceding transition, and the remaining difficult transitions will become spaced evenly over the habitable interval.

If all of the transition rates become more rapid, so that the expected transition times are approximately equal to 1/*n*th the lifetime of Earth (*λ*_*i*_ = *n*/*L*), the distributions will all skew more towards the start of Earth’s lifetime. However, there will still remain a substantial probability that the final few steps will occur in the latter half of Earth’s lifetime (figure 2*e*). When all rates are rapid enough to be expected well within Earth’s lifetime (*λ*_*i*_ = 10*n*/*L*), the transition distributions converge to a sum of exponential distributions (figure 2*f*). Since the probability of successfully achieving intelligence within the Earth’s lifetime is so high with such parameters, the need to condition on the final transition occurring before the end of Earth becomes redundant and the distributions converge to the unconditional distributions without observation selection effects.

### (a) Catastrophes on evolutionary transitions

With an understanding of how the model behaves without any catastrophes or evolutionary dead ends, we can now investigate these effects on evolutionary transitions.

**Figure 3.**
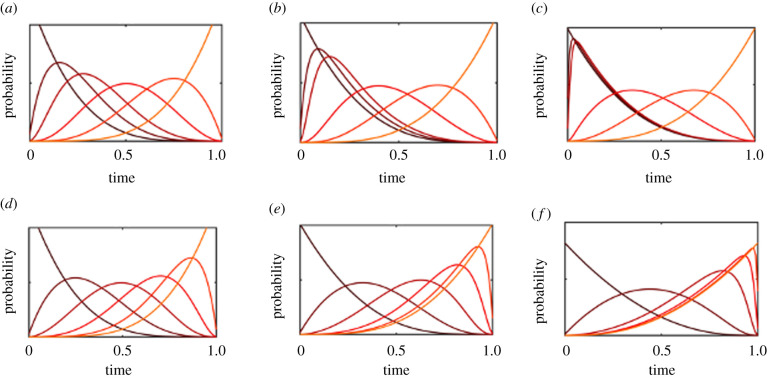
A model of six critical steps with catastrophes occurring at a constant rate. The first row describes a model in which catastrophes affect the first two transitions, and sets the chain back to the 0th state. Rates of catastrophe move from *μ* = 5 (*a*), to *μ* = 20 (*b*) and *μ* = 100 (*c*). The second row describes a model in which life becomes increasingly vulnerable to catastrophes as it becomes more complex, with the final transition vulnerable to a catastrophe with rates of *μ* = 5 (*d*) to *μ* = 20 (*e*) and *μ* = 100 (*f*). The two transitions preceding the final one have catastrophe rates one-third and one-ninth of the final catastrophe rate (for the 5th and 4th transition, respectively). Note that the spacing between evolutionary transitions decreases over time. (Online version in colour.)

Results for both the early and late catastrophe models are shown in figure 3. Our key finding is that critical steps that are vulnerable to catastrophes will occur on a faster time scale that is driven by the rate of catastrophe rather than the rate of evolutionary transition. The higher the catastrophe rate, the faster the vulnerable transitions. If multiple transitions are vulnerable to a catastrophe, they can become tightly clustered and occur on a short time scale.

This can be seen most clearly in the second type of model that represents fragile early life, shown in figure 3*a*–*c*. Here, we show results for a six step model, where the first two transitions are vulnerable to a catastrophe but the subsequent four transitions are immune. All evolutionary transition rates are set to *λ* = 10^−3^, and we assume that a catastrophe reverts the chain to the 0th state, representing a disaster that entirely destroys fragile early life. When the catastrophe rate is low (*μ* = 5), the effect is minor and the first three transitions will occur slightly faster than the baseline model without catastrophes. However, as the catastrophe rate increases (to *μ* = 20 or *μ* = 100), the first transitions will occur back-to-back in a tight cluster. Moreover, the rapid early transitions provide more time for the subsequent transitions to occur, so that the transitions which are immune to catastrophes will still occur in an evenly spaced manner across the remaining habitable lifetime of the planet. In a six step model with the first two transitions vulnerable and a high enough catastrophe rate, the probability density functions for the transition times closely resemble those of a model with only four critical steps (where the vulnerable steps occur almost simultaneously with the first non-vulnerable step).

For the model where more complex forms of life are more vulnerable to catastrophes with *λ* = 10^−3^, *μ*_1_ = *μ*_3_/9 and *μ*_2_ = *μ*_3_/3 produces the results in figure 3*d*–*f* with *μ*_3_ = 5 (*d*), *μ*_3_ = 20 (*e*) and *μ*_3_ = 100 (*f*). As before, when the catastrophe rate is exceptionally high, the transitions that are vulnerable will become clustered closely together and occur right before the final transition. More interesting is what occurs at intermediate catastrophe rates. Since later transitions are vulnerable to more catastrophes, the catastrophe rate effectively increases with each critical step. As each critical transition is pitched against the catastrophe rate it is subjected to, the speed of each transition will be greater than the previous one. This leads to a general acceleration in the frequency of critical steps, so that any intelligent observers will see an evolutionary history with many more critical steps occurring in their recent history as opposed to their ancient history. The spacing between transitions will also decrease over time.

### (b) Niche incumbency

We now investigate the role evolutionary dead ends (table 1). An evolutionary transition can be precluded if a transition to the dead end occurs before a transition to the next step towards intelligent life. We assume that transitions to an evolutionary dead end occur at constant rate *θ*.

**Figure 4.**
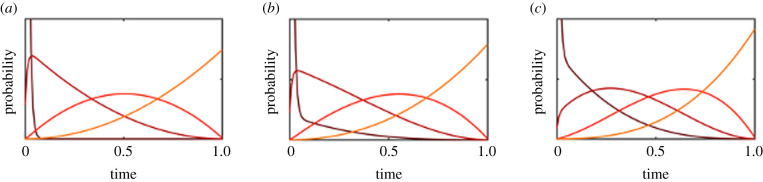
(*a*) A model with four transitions, where the first transition is competing with an evolutionary dead end with rate *θ* = 100. (*b*,*c*) Models indicating a catastrophe that can undo the evolutionary dead end with rate *μ* = 1 (*b*) and *μ* = 10 (*c*). Without catastrophes, this is close to the situation where the first transition has a rate *λ*_1_ = 100, but as the catastrophe rate increases, the effect of evolutionary dead ends decreases. (Online version in colour.)

As with the catastrophes, the ultimate effect of these evolutionary dead ends is to accelerate any difficult transitions that are competing with an evolutionary dead end. The results are shown in figure 4. In a model with four critical steps where the final step is competing with an evolutionary dead end, the final step will tend to dovetail closely with the third step. If the first two transitions are competing with evolutionary dead ends, they will occur much more rapidly.

The reason is similar to why catastrophes can accelerate a critical step. The critical step needs to occur before falling into an evolutionary dead end, and so is therefore dictated by the time scales associated with the evolutionary dead end rather than the time scales associated with the remaining habitable time.

When evolutionary dead ends can be reversed, an evolutionary history that eventually leads to intelligent life may spend time in that dead end state, and increases in the catastrophe rate will reduce the probability of spending a substantial amount of time in the dead end state. If the catastrophe rate becomes greater than the transition rate compared to evolutionary dead ends (i.e. *μ* ≫ *θ*), then the evolutionary dead ends will cease to play a large role and the critical steps will become evenly spaced throughout the Earth’s lifetime (figure 4). This only holds true if the catastrophe affects the evolutionary dead ends but not the critical steps. If the catastrophe affects both the evolutionary dead ends and the critical step then increasing the catastrophe rate will reduce the amount of time spent in the vulnerable states.

### (c) Neocatastrophism hypothesis

**Figure 5.**
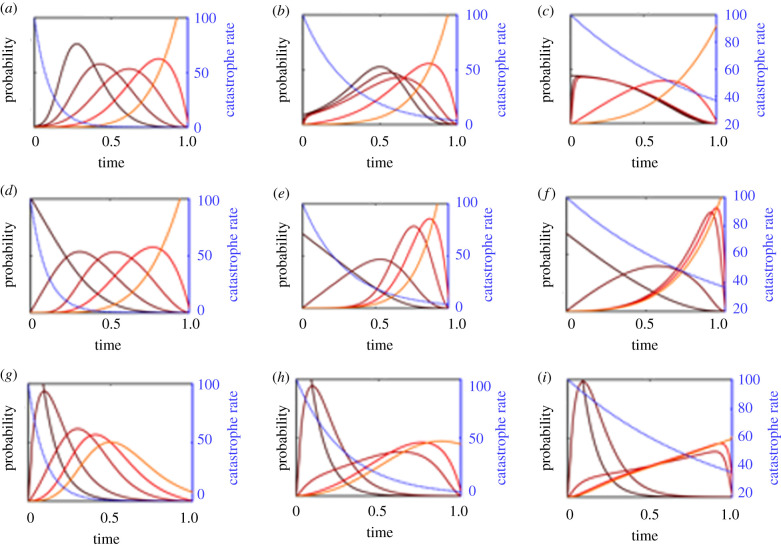
Model with five critical steps and a catastrophe rate that declines exponentially over time as the function $\mu (0)\;e^{-t/t_{\gamma }}$. *μ*(0) is set to 100 for all models, and $t_{\gamma }=0.1$ (*a*,*d*,*g*), 0.3 (*b*,*e*,*h*) and 1 (*c*,*f*,*i*), resulting in a slower decline of rates as one moves from left to right. In the first row, the first two transitions are vulnerable to catastrophes, with each catastrophe sending the chain to the 0th state. In the bottom two rows, the final three transitions are vulnerable, with each catastrophe sending the chain back to the 2nd state. In the first two rows, evolutionary transitions have rates of *λ* = 10^−3^, in the bottom row, *λ* = 10, representing fast rates. (Online version in colour.)

To investigate the neocatastrophism hypothesis, we begin by setting the initial catastrophe rate to *μ*(0) = 100, and varying the decay rate parameter with values $t_{\gamma }=0.1$, 0.3 and 1. The results are shown in figure 5. These parameter choices are selected to highlight different model behaviours. Rate estimates for biologically meaningful galaxy-wide catastrophies (such as gamma-ray bursts) vary substantially; ranging from once every 170 Myr for mass-extinction-level events [21] to once every 13.8 Gyr (as calculated from a Poisson model in [22] using analysis from [23]). Gamma-ray bursts that would sterilize the biosphere are thought to be substantially more rare, with probability estimates below 10^−7^ per Gyr [24].

We analyse the first neocatastrophism model, in which the first two transitions are vulnerable to catastrophes. When the catastrophe rate declines quickly, these early transitions will be delayed until the rate falls to a low enough level (to an average of about one catastrophe every 5% of the Earth’s lifetime). Evolutionary transitions are then evenly spaced out throughout the remainder of the Earth’s habitable lifetime (figure 5*a*). When the catastrophe rate declines more slowly, the early vulnerable transitions can be pushed even further back, and the time between vulnerable transitions is reduced (figure 5*b*). However, when the catastrophe rate declines so slowly that the rate remains high throughout Earth’s entire lifetime, the first three transitions will occur in a tight cluster with higher probabilities towards the beginning of Earth’s lifetime (figure 5*c*). This can be explained by the balance between the relative improbability of vulnerable transitions occurring early (when the catastrophe rate is high), as opposed to the improbability of final transitions occurring in the time remaining. When the decline in catastrophe rate is fast, the vulnerable transitions can occur later on when the catastrophe rate is low, and still leave time for subsequent transitions. When the decline in the catastrophe rate is slow, the increased probability that comes with delayed vulnerable transitions is not enough to offset the time needed for the later transitions to occur.

When the first few transitions are unaffected by the catastrophes, but the final transitions are, the dynamics can become substantially different. The first obvious difference is that the first few transitions can occur even when the catastrophe rate is at its highest, and so therefore are not prevented from occurring early. When the catastrophe rate declines quickly, this results in a model that is hard to distinguish from a standard critical steps model (figure 5*d*), since the catastrophe rate will be negligible by the time the vulnerable transitions start to become possible. When the catastrophe rate declines more slowly, a more interesting pattern emerges, with hard evolutionary transitions occurring more towards the end of the interval, in a more tightly spaced manner (figure 5*e*). Moreover, the transitions that are unaffected by catastrophes become spaced further apart during the high-catastrophe-rate period. When the catastrophe rate declines extremely slowly, the model becomes similar to one with constant catastrophe rates, with a very tight cluster of transitions occurring towards the end of the habitable interval (figure 5*f*).

It is important to note that the models we have discussed so far represent a combination of the neocatastrophism hypothesis with the Carter argument, since we still assume that intelligent life requires a number of critical steps, each of which has an expected transition time greatly exceeding the lifetime of Earth. This is in contrast to the neocatastrophism hypothesis as a strict alternative to the Carter argument, which would describe a situation in which the time scales associated with intelligent life are substantially shorter, so that the primary bottleneck is the time between catastrophes rather than the total time of planetary habitability.

We represent the strict neocatastrophism hypothesis (that there were no critical steps, but rather catastrophes that delayed intelligent life), by adjusting the previous five step model to have fast evolutionary transition rates (*λ* = 10, corresponding to each transition taking roughly 10% of the Earth’s lifetime). In the absence of catastrophes, intelligence would therefore have a high probability of emerging within Earth’s lifetime (about 97%, derived from the Erlang cumulative distribution function evaluated with rate *λ* and shape parameter *n*, giving a mean of *λn*). We then investigate this by adding catastrophes, assuming (as previously) that the first two transitions are unaffected by catastrophes but the final transitions are affected.

As before, the presence of catastrophes will delay the onset of the final evolutionary transitions. However, there are two noticeable differences with this model and the previous one that had hard critical steps. First, the transitions unaffected by catastrophes occur much earlier, and second, the variance in the timing of the vulnerable transitions is higher (figure 5*g*–*i*). The first difference (of early transitions occurring rapidly) is a straightforward consequence of fast transition rates and the fact that these transitions are unaffected by catastrophes. The second difference (of increased variance in the timing of the remaining transitions) is in part due to having more time left since the initial transitions occur quickly. Another key driver is that since the transition rates are more rapid, there is a higher probability that all transitions can occur before a catastrophe. Changes in the catastrophe rate have a smaller impact on the relative probability of making the transitions earlier or later. This results in a substantially greater probability of intelligent life emerging earlier in Earth’s history.

## 4. Discussion

Here, we have shown that by extending and generalizing Carter’s original model to include catastrophes or evolutionary dead ends, the timing of critical steps can become accelerated, and the time between critical steps compressed. This opens up new hypotheses about whether some critical steps and hence evolutionary transitions have been overlooked.

### (a) Role of catastrophes on evolutionary transitions

Our first new hypothesis is that evolutionary transitions accelerate in the face of catastrophes. Watson [9] argued that, since the first three evolutionary transitions described by [5] had occurred close together, it was unlikely that more than one of them had an associated time scale longer than the habitability of Earth. Conversely, our model suggests that all three associated time scales could exceed the lifetime of Earth and still be consistent with the data, so long as all three transitions were needed to produce life capable of surviving a stochastic event with a rapid enough rate.

The reason for these accelerated transitions is simple. Instead of just racing against the lifetime of the planet, the vulnerable transitions are also racing against the possibility of being undone by a catastrophe. The faster the catastrophes happen, the faster the vulnerable transitions must occur in order to avoid such a fate. Once a resilient state has been reached, then the remaining transitions are racing only against the lifetime of the planet, and will conform to the original critical steps model by being evenly spaced out throughout the remaining habitable time left.

A second intriguing hypothesis opened up by our work is that although life on Earth has existed for over 3.5 Gyr [25], a staggering amount of complexity has increased in the final 15% of that time [26], with major changes in the biota of Earth including the Cambrian Explosion (<550 Ma [27]), the first plants on land (<520 Ma, [28]), the first vertebrates on land (<350 Ma [29]), greater encephalization (<10 Ma [30]), and the emergence of hierarchical language and intelligence itself (<1 Ma [31]). Although past models of critical steps suggested that at most one or two of these transitions (or others around the same time) could be critical steps, the possibility that catastrophes could undo these levels of complexity may account for the sense of accelerating transitions. The catastrophes that could undo all encephalized lineages are a small subset of the catastrophes that could destroy all land vertebrates, which is in turn a subset of the catastrophes that would destroy all complex multicellular life. This in turn means that the effective catastrophe rate will increase with each step towards intelligence, and thus that each critical step will occur more rapidly than the previous one. This acceleration comes not due to the actual realization of a catastrophe, but rather the risk of a catastrophe driving the rates faster when subject to observation selection effects. However, we have also shown a possible way that catastrophes that occur could accelerate evolution, in part related to the theories of punctuated equilibrium versus gradualism [32].

One limitation of the critical steps model is that we divide the evolution of life into a small number of improbable transitions (also see [12]) operating within the finite lifetime of the star. However, it is evident that these evolutionary transitions may not necessarily be independent of planetary processes, as originally hypothesized (by Carter [3]). For instance, Scharf & Cronin [33] suggest that greater understanding of different structures in solar systems, such as the varying probabilities that exoplanets share material, might alter parameter space under which biological evolution is initiated and/or accelerates. Elsewhere, we expand on these assumptions and emphasize limitations to the critical steps model [12] such as ‘fixed clock’ requirements to transitions (e.g. such as oxygen build-up). Understanding these limitations further and the implications of catastrophes driving evolutionary transitions will involve relaxing key assumptions of the critical steps model such as exponential waiting times for transitions. Furthermore, assessing evolutionary acceleration simply in terms of morphological complexity (e.g. [26]) is most likely fraught with difficulties as this neglects the necessary evolutionary development of molecular and intracellular complexity (that is extremely poorly preserved in early geological records). Developing these Markov chain approaches further to investigate this under a wide breadth of transition probability frameworks coupled with richer planetary, geological and biological details will be an exciting area for future work.

### (b) Niche incumbency

Our work suggests a third hypothesis, namely that critical steps could occur more rapidly if they are competing against a faster evolutionary transition that leads to a dead end. Although speculative, evidence suggests that while alternatives to the four-nucleotide genetic coding systems (e.g. [34]) or alternatives to the three-base codon systems (e.g. [35]) may have been more readily derived from the available chemistry, these alternatives might have had slower downstream rates of evolution or limits to the amount of biological complexity that could be supported [36]. Within this hypothesis, that evolutionary state and trajectory can influence niche incumbency, competition and exclusion warrants further investigation.

### (c) Neocatastrophism and the Carter argument

Furthermore, using our evolutionary transition model framework, we have investigated different hypothesis for the scarcity of intelligent life. Carter argues (through simple probability calculations) that intelligent life is rare due to a number of highly improbable evolutionary transitions in the lifetime of a star (see also [12]).

Given that the neocatastrophism hypothesis has been suggested as an alternative to the Carter argument, it is important to compare these models and their predictions in light of our findings. The Carter argument predicts that a small set of critical steps were required for intelligent life on Earth, and that these steps will be spaced out roughly evenly across Earth’s lifetime. It also predicts that intelligent life is exceptionally rare, but when it does emerge it does so in the late stages of a planet’s lifetime. While the neocatastrophism hypothesis posulates simultaneous emergence of complex life late in the lifetime of a star due to the declining effects of catastrophes (such as gamma-ray bursts) across galaxies.

If we reject the Carter argument and instead rely on our neocatastrophism model to explain the apparent lifelessness of our galaxy, a number of other predictions emerge that actually seem to fit poorly with data. The first is that any evolutionary transitions that are unaffected by the catastrophes in question should occur rapidly and early in a planet’s lifetime. In our model, this is seen with the first few transitions happening quickly. However, given that galaxy-wide catastrophes (such as gamma-ray bursts) will primarily affect life on land or shallow water [21], a declining rate of these events fails to explain why it took so long for other evolutionary transitions that occurred within the oceans. Eukaryotes took well over 1 billion years to emerge [37], and it took another billion years for eukaryotic life to develop into the complex multicellular life found in the Cambrian Explosion [38]. The Carter argument provides a parsimonious explanation for why some of these evolutionary transitions may have taken such a long fraction of Earth’s history, beyond explaining why intelligent life emerged in the late stages of Earth’s history. In summary, explaining why intelligent life emerged on roughly the same time scale as the lifetime of Earth is insufficient for an alternative hypothesis to the Carter argument. Any alternative hypothesis would also need to explain why a number of other evolutionary transitions seem to have occurred on roughly the same time scale as the lifetime of Earth, and the hypothesis that catastrophes have stunted biological development on Earth fails to explain this.

The high variance in the emergence time of intelligent life under the neocatastrophism model also undermines the argument that intelligent life would originate concurrently across the galaxy once catastrophes have fallen to a low enough rate. Instead, we would still expect some planets to support intelligent life hundreds of millions of years before others. This is because although most series of vulnerable evolutionary transitions occur only once the catastrophe rate is low, a substantial minority of transition sequences can occur more rapidly than usual, occurring even when the catastrophe rate is higher.

Finally, if the primary bottleneck to intelligent life truly was a high catastrophe rate which declined over time, we should perhaps expect to see more dramatic evidence of this in the fossil record. For example, if vertebrates on land had evolved early in Earth’s history, followed by hundreds of cycles of extinction and gradual recolonization of land, this would be strong evidence that the extinctions were preventing life from fully exploring the niches that could beget intelligence. One could imagine an even more dramatic fossil record exhibiting an increase in animal brain size over time that was regularly cut short by each catastrophe, before eventually reaching the size of a human brain. However, we instead see that vertebrate life on land did not emerge until very recently (340 Ma) [29], and that animal body sizes were large enough to have had human-sized brains for hundreds of millions of years [39]. Although catastrophes may have played a role in the subsequent evolution and radiation of land vertebrates, it is hard to see how catastrophes would have delayed the emergence or have prevented intelligence from originating over 300 Ma.

We conclude that the neocatastrophism hypothesis fails on two fronts. First, although it can explain a delay for transitions that are vulnerable to catastrophes, it fails to explain the delay for transitions that could occur in environments less susceptible to such catastrophes, such as eukaryotic life or the Cambrian explosion. Second, it fails to explain the absence of intelligent civilizations elsewhere in our galaxy. The high variance in the arrival time of intelligent life under a neocatastrophsim model would instead suggest that a non-trivial fraction of intelligent life will emerge hundreds of millions of years before ourselves, contrary to the evidence we see. Conversely, the Carter argument resolves both of these problems: the long transition times and the absence of intelligent life in our galaxy can both be explained by a series of exceptionally difficult evolutionary transitions.

Complex, intelligent life emerged late in Earth’s history, after a series of evolutionary transitions occurring over billions of years. Rather than equally spaced evolutionary transitions to explain complex life, if increasing biological complexity also leads to increasing vulnerability to catastrophes, the final transitions will exhibit an accelerating pattern, with each critical step occurring more rapidly than the previous one. This is a striking result, given how much biological complexity has increased in the past 500 Myr.

## Data Availability

All scripts used in this study are openly available at https://osf.io/4xrb9/. Further information is available in the electronic supplementary material [40].
